# Comparison of initial patient setup accuracy between surface imaging and three point localization: A retrospective analysis

**DOI:** 10.1002/acm2.12183

**Published:** 2017-09-13

**Authors:** Dennis N. Stanley, Kristen A. McConnell, Neil Kirby, Alonso N. Gutiérrez, Nikos Papanikolaou, Karl Rasmussen

**Affiliations:** ^1^ Radiation Oncology ‐ Medical Physics University of Texas Health Science Center San Antonio San Antonio TX USA; ^2^ Miami Cancer Institute Baptist Hospital Miami FL USA

**Keywords:** C‐RAD CatalystHD, patient setup, SGRT, subcutaneous tattoos, surface imaging

## Abstract

**Purpose:**

Historically, the process of positioning a patient prior to imaging verification used a set of permanent patient marks, or tattoos, placed subcutaneously. After aligning to these tattoos, plan specific shifts are applied and the position is verified with imaging, such as cone‐beam computed tomography (CBCT). Due to a variety of factors, these marks may deviate from the desired position or it may be hard to align the patient to these marks. Surface‐based imaging systems are an alternative method of verifying initial positioning with the entire skin surface instead of tattoos. The aim of this study was to retrospectively compare the CBCT‐based 3D corrections of patients initially positioned with tattoos against those positioned with the C‐RAD CatalystHD surface imager system.

**Methods:**

A total of 6000 individual fractions (600–900 per site per method) were randomly selected and the post‐CBCT 3D corrections were calculated and recorded. For both positioning methods, four common treatment site combinations were evaluated: pelvis/lower extremities, abdomen, chest/upper extremities, and breast. Statistical differences were evaluated using a paired sample Wilcoxon signed‐rank test with significance level of <0.01.

**Results:**

The average magnitudes of the 3D shift vectors for tattoos were 0.9 ± 0.4 cm, 1.0 ± 0.5 cm, 0.9 ± 0.6 cm and 1.4 ± 0.7 cm for the pelvis/lower extremities, abdomen, chest/upper extremities and breast, respectively. For the CatalystHD, the average magnitude of the 3D shifts for the pelvis/lower extremities, abdomen, chest/upper extremities and breast were 0.6 ± 0.3 cm, 0.5 ± 0.3 cm, 0.5 ± 0.3 cm and 0.6 ± 0.2 cm, respectively. Statistically significant differences (*P *<* *0.01) in the 3D shift vectors were found for all four sites.

**Conclusion:**

This study shows that the overall 3D shift corrections for patients initially aligned with the C‐RAD CatalystHD were significantly smaller than those aligned with subcutaneous tattoos. Surface imaging systems can be considered a viable option for initial patient setup and may be preferable to permanent marks for specific clinics and patients.

## 1 INTRODUCTION

Historically, the process of positioning a patient prior to imaging verification used a set of permanent patient marks, or tattoos, placed subcutaneously. These subcutaneous tattoos are traditionally placed medially and on both lateral sides using a permanent ink injected at a 30° angle perpendicular to the surface of the skin to a depth of 1–2 mm.^1^, ^2^ This configuration allows for a three point localization of the initial isocenter. After aligning to these tattoos, plan specific shifts are applied, and, often, the position is verified with x‐ray volumetric imaging.^3^ Due to a variety of factors, including changes in body habitus, localized radiation induced swelling/shrinking, and a significant time difference between the placing of the marks and treatment, these marks may deviate from the desired position or it may be hard to align the patient to these marks.

Additionally, as overall patient survival continues to increase, many patients are requesting not to have a permanent tattoo placed, especially in pediatrics and breast cases.^4^, ^5^ A publication from Crow and Allen in 2010 detailed the psychosocial impacts of radiation tattooing for breast cancer patients and specifically the long‐lasting effects and poorly documented psychological effects that can happen long after treatment.^5^ Of the women who were surveyed many found “the tattoos as compounding the trials of dealing with significant physical changes to their breasts and bodies arising from diagnosis and treatment.”^5^ The tattoos serve as a lasting and constant reminder of cancer treatment long after treatments have finished. While some patients may see these as necessary inconvenience, clinical providers should be considerate of the patient experience, especially when an alternative method may have a reduced long term psychological effect. Along with the psychological impact, an increased prevalence of patients who have an allergy to many of the specific inks used in subcutaneous marks has been reported.^6^, ^7^ While these situations may be comparatively rare, it is important to have a reliable and accurate method of pre‐positioning verification. With the increasing use of nonionizing surface‐based imaging systems, an alternative method of verifying initial positioning exists that relies on the entire skin surface instead of tattoos. The aim of this study was to retrospectively compare the 3D corrections of patients positioned with tattoos and with C‐RAD CatalystHD.

## 2 MATERIALS AND METHODS

### 2.A C‐RAD CatalystHD

The C‐RAD CatalystHD (C‐RAD, Uppsala, Sweden) is a ceiling‐mounted three camera nonionizing optical based imaging system capable of monitoring patient setup and positioning, intra‐fraction and inter‐fraction motion detection, and respiratory gating. The CatalystHD has a scanning volume (X*Y*Z) of a 800 × 1300 × 700 mm with a scan speed of 220 complete 3D surfaces per second. The cameras are mounted at fixed angles so that localization and visualization of the patient are maintained for every gantry location by projecting specific light patterns of a known wavelength (405 nm (near‐invisible violet), 528 nm (green) and 624 nm (red)) onto the patients’ skin. The CatalystHD algorithm then matches the light pattern that is projected onto the patients’ surface to a reference surface acquired previously (either from the reference CT or from baseline volume scan done prior to treatment). Based on the two acquired images of the patient surface, the CatalystHD algorithm provides the patient positioning errors and provides a correction of target positioning expressed in the six degrees of freedom.

### 2.B Methods

Over a 24 month period, a total of 6000 individual fractions were initially aligned using either the CatalystHD or the traditional three point subcutaneous marks. Between 600 and 900 individual fractions per site per method were randomly selected and recorded for all patients and fractions. For the three point subcutaneous mark positioning technique, patients were aligned to the marked locations and the planned shifts were implemented manually by the therapists. For the CatalystHD positioning technique, patients were imaged, shifts were calculated, and sent to the linear accelerator for automatic adjustment. For both initial positioning methods, a kV‐CBCT was acquired immediately following to adjust for any residual corrections. The anatomical goal for these alignment corrections varied widely across all evaluated fractions but were consistent within each patient regardless of initial setup technique. Four common treatment site combinations were evaluated for both positioning methods: pelvis/lower extremities, abdomen, chest/upper extremities, and breast. Statistical differences of the residual corrections were evaluated using a paired sample Wilcoxon signed rank test with significance level of <0.01. All evaluated patients had the ability to be initially positioned with either setup technique and the specific choice of setup technique was at the discretion of the designated setup therapist, which rotated weekly. All of the patients included in this data analysis had setups from both techniques, to avoid potential bias from patients that would have a clinical preference for only one setup technique. Additionally, therapists either setup with the traditional three point subcutaneous marks or the surface imaging system, but not both.

## 3 RESULTS

**Figure 1:**
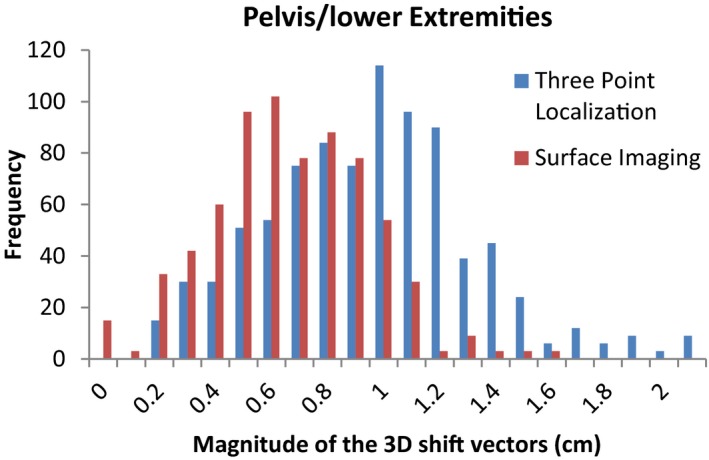
Cumulative histograms showing the pre‐CBCT 3D corrections for a traditional three point localization and surface imaging techniques for the pelvis/lower extremities.

**Figure 2:**
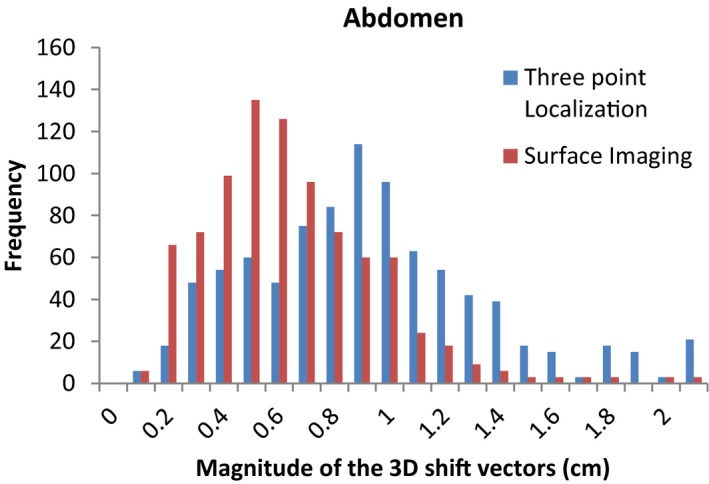
Cumulative histograms showing the pre‐CBCT 3D corrections for a traditional three point localization and surface imaging techniques for the abdomen.

**Figure 3:**
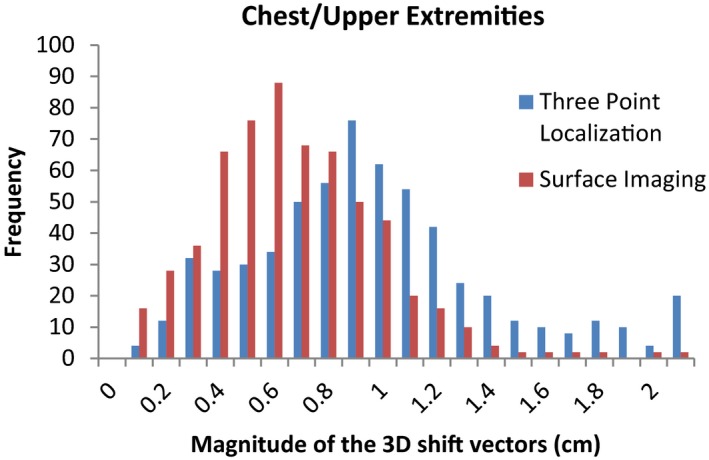
Cumulative histograms showing the pre‐CBCT 3D corrections for a traditional three point localization and surface imaging techniques for the chest/upper extremities.

**Figure 4:**
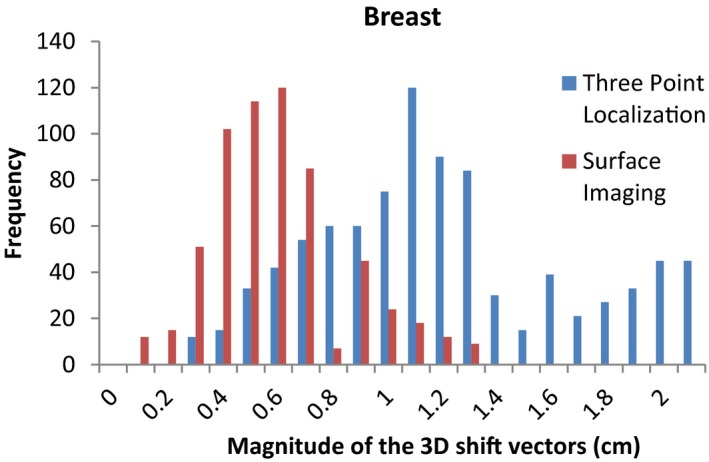
Cumulative histograms showing the pre‐CBCT 3D corrections for a traditional three point localization and surface imaging techniques for the breast.

The average magnitudes of the post‐CBCT 3D shift vectors and the standard deviations for the traditional three point localization using subcutaneous tattoos and localization using the surface imaging technique are listed in Table 1. Statistically significant differences (*P *< 0.01) in the post‐CBCT 3D shift vectors were found for all four sites. Figures 1, 2, 3, 4 show histograms of the magnitude of the 3D shift vectors for both methods for all four site combinations.

**Table 1:** Summary of post‐CBCT 3D corrections calculated averages and standard deviations for a traditional three point localization with subcutaneous tattoos and surface imaging techniques

	Three point localization		Surface imaging	
	Average(cm)	*σ*(cm)	Average(cm)	*σ*(cm)
Pelvis/lower extremities	0.9	0.4	0.6	0.3
Abdomen	1.0	0.5	0.5	0.3
Chest/upper extremities	0.9	0.6	0.5	0.3
Breast	1.4	0.7	0.6	0.2

## 4 DISCUSSION

Historically, subcutaneous marks served as the primary (and often the only) method of initial setup up for patients prior to verification. Although, with the growth of the modern state‐of‐the‐art image guidance systems for use in Image Guided Radiation Therapy (IGRT), including surface imaging systems, the dependence on and relevance of these marks has been drastically reduced.^8^, ^9^ With current standards of care, where volumetric imaging is employed prior to treatment for 3D setup corrections, subcutaneous marks serve as a relic of the pre‐volumetric imaging standards or positioning. These permanent tattoos, in the most basic sense, are “stationary” landmarks on an elastic body organ whose elasticity is drastically changed during radiation therapy.^10^ It is not uncommon for therapists to stretch and pull portions of the skin with these marks to make marks properly align. This ability to manually manipulate marks highlights the need for an alternative method. For treatments like lung Stereotactic Body Radiation Therapy (SBRT) and the treatment of breast cancer with deep inspiration breath hold, where surface imaging technologies are already being utilized, the question becomes are tattoos necessary?^11^ Clinically, regardless of the pre‐imaging verification setup method, it is assumed that patients are being positioned correctly due to the 3D volumetric imaging. It is important and should be noted that this study is not advocating for a complete removal of subcutaneous tattoos in radiation therapy but rather to evaluate an alternative solution for primary setup that may be preferable for specific clinics and patients.

## 5 CONCLUSION

This study shows that the overall 3D shift corrections for patients initially aligned with the C‐RAD CatalystHD were significantly smaller than those aligned with subcutaneous tattoos. Surface imaging systems should be considered a viable option for initial patient setup and may be preferable to permanent marks for specific clinics and patients.

## CONFLICT OF INTEREST

None of the authors have a conflict of interest to report.
